# Impact of maxillary sinus augmentation on oral health-related quality of life

**DOI:** 10.1186/s40729-017-0072-8

**Published:** 2017-03-28

**Authors:** E. Schiegnitz, P. W. Kämmerer, K. Sagheb, A. J. Wendt, A. Pabst, B. Al-Nawas, M. O. Klein

**Affiliations:** 1 Department of Oral and Maxillofacial Surgery, Plastic Surgery, University Medical Centre of the Johannes Gutenberg-University, Augustusplatz 2, 55131 Mainz, Germany; 2 Department of Oral and Maxillofacial Surgery, Plastic Surgery, University of Rostock, Rostock, Germany; 3 Department of Prosthodontics, University of Hamburg-Eppendorf, Hamburg, Germany; 4 Oral and Maxillofacial Surgery, Private Praxis, Düsseldorf, Germany

**Keywords:** Dental implant, Maxillary sinus augmentation, Oral health-related quality of life

## Abstract

**Background:**

The aim of this study was to measure the oral health-related quality of life (OHRQoL) after maxillary sinus augmentation to determine the physical and psychological impact of this procedure for the patient.

**Methods:**

Three hundred sixteen patients treated with an external or internal maxillary sinus augmentation and a total of 863 implants in the Department of Oral and Maxillofacial Surgery, Johannes Gutenberg University, Mainz, Germany, between July 2002 and December 2007 were included in this retrospective study. Total implant survival was assessed. Completion of a modified 26-item version of the Oral Health Impact Profile (OHIP-G) for assessing the oral health-related quality of life before and after the treatment was asked for. Subcategories were (1) functional limitations, (2) physical and psychological disabilities, and (3) complaints due to the surgical procedure. In 53 patients available for clinical follow-up examination, assessment of soft tissue parameters was performed.

**Results:**

After an average time in situ of 41.2 ± 27 months (3.4 years), the in situ rate was 95.4%. One-year survival rate and five-year survival rate according to Kaplan Meier were 95.4 and 94.4%. Concerning functional limitations, significant better values for OHRQoL after sinus augmentation procedure than before the treatment (*p* < 0.001) were seen. In the subcategory physical and psychological disabilities, all questions had significant better values after the sinus lift (*p* < 0.001). Concerning complaints due to the surgical procedure, mean total scores were 5.1 ± 5.4 pre-operative, 6.9 ± 6.1 (0–31) post-operative, and 2.4 ± 3.7 recently. This meant a significant difference between “pre-operative” vs. “post-operative” (*p* = 0.003), “pre-operative” vs. “recently” (*p* < 0.001), and “post-operative” vs. “recently” (*p* < 0.001). Concerning the influence of implant indication, edentulous patients showed the most distinct improvement after the procedure. Clinical assessment showed stable soft tissue parameters.

**Conclusions:**

Evaluation of OHRQoL after sinus augmentation showed a significant improvement indicating a remarkable benefit for the patients through this procedure.

## Background

Rehabilitation of completely and partial edentulous patients with dental implants has proved to be a safe and predictable procedure [1–3]. However, reduced bone height and the proximity of the maxillary sinus are challenging limitations for dental implant placement in the posterior maxilla [3]. Besides the use of short and tilted implants [4], one of the most frequently used surgical techniques for gaining adequate bone height in the posterior maxilla is external or internal maxillary sinus floor elevation. Several systematic reviews of the literature showed high overall implant survival rates well beyond 90% for sinus floor evaluation [1, 5, 6]. In addition, a recent Cochrane Systematic review including 18 randomized controlled trials (RCT) confirmed these high survival results [7]. However, the patients’ perspective was mostly not appropriately taken into account in these analyses, although patient satisfaction presents one of the most essential objectives to obtain in oral rehabilitation [8, 9]. Hence, the question remains if the patients benefit from the sinus elevation procedures regarding their oral health-related quality of life (OHRQoL). However, studies evaluating the patient’s perception after sinus elevation are very rare.

OHRQoL is a complex patient-centered concept that observes the impact of oral states of health on the well-being of individuals and society and assesses the effects of dental interventions [10, 11]. Different items like age, alcohol or tobacco habits, dental diseases, dentition, tooth loss, and condition of prosthesis affect OHRQoL [10, 12]. In addition, sociodemographic, financial, cultural, educational, psychological, and dietary factors have to be considered [13]*.* These patient-oriented outcomes can be examined using several different tools, including the Oral Health Impact Profile (OHIP), which is the most widely applied measure [14–16]. The OHIP represents a self-reported questionnaire on OHRQoL consisting of 49 questions under seven subscales [17]. The OHIP was translated to several different languages like German, Spanish, and Chinese, and shortened versions like OHIP-14 were introduced to reduce the response time [18–20]. The validity, sensitivity, and specificity of OHIP as a measuring instrument were validated in a huge variety of settings [21–23].

In conclusion, little information is available about patient’s perception of sinus augmentation procedures. The aim of the present study was to assess whether sinus augmentation procedures together with implant placement and prosthetic rehabilitation improve quality of life in dental patients using a modified German OHIP and to examine the survival rates after this procedure.

## Methods

### Study design and subjects

**Fig. 1:**
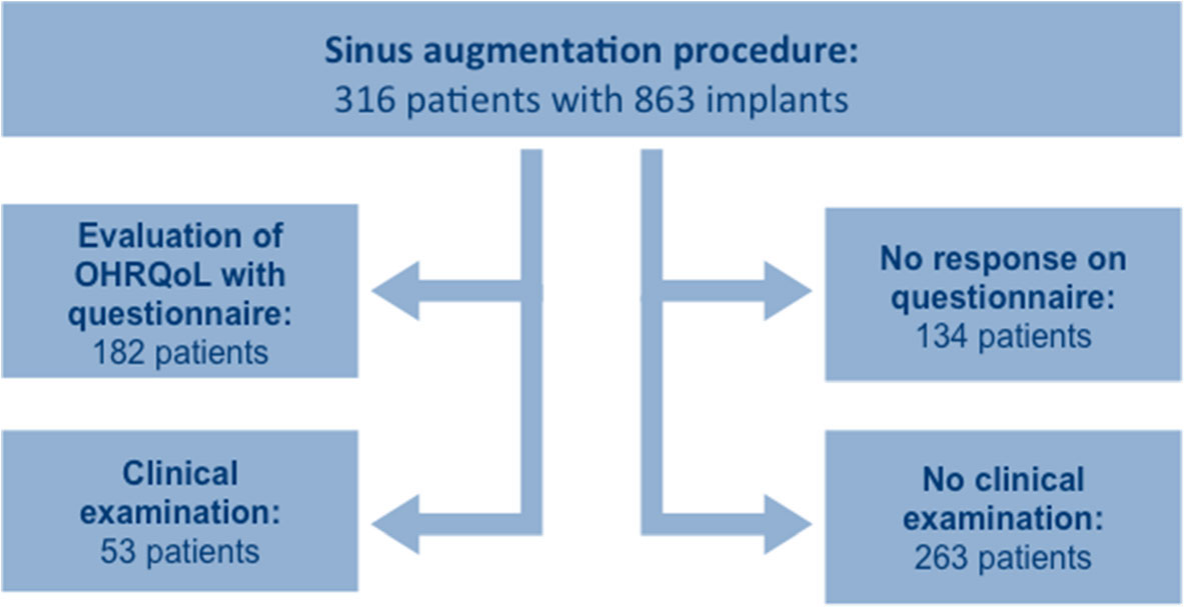
Flow chart of patients included in the study

This retrospective study addresses the oral health-related quality of life after maxillary sinus augmentation. Therefore, all patients that received an implantation after maxillary sinus augmentation in the Department of Oral and Maxillofacial Surgery of the University Medical Centre Mainz, Germany, between July 2002 and December 2007 were included in this study. There were no specific exclusion criteria. In this time period, 863 implants in 316 patients after sinus augmentation were inserted. One hundred forty-two of these patients (44.9%) were men and 174 (55.1%) women. Mean age of men was 57.4 years and mean age of women 55.2 years. Fifty-three patients (33 women and 8 men), with 157 dental implants remaining in situ, attended a clinical follow-up examination (Fig. 1). For these patients, plaque index, gingival index, probing depth, and width of keratinized mucosa were evaluated. The retrospective data analysis was conducted in accordance with the Helsinki Declaration of 1975, as revised in 2008, and all patients signed an informed consent. After consulting the local ethic committee, the decision was that due to the retrospective character of this study with no additional data acquisition, no ethical approval was needed according to the hospital laws of the appropriate state (Landeskrankenhausgesetz Rhineland Palatinate, Germany).

### Measurement of OHRQoL

For evaluation of OHRQoL after sinus lift procedures, a modified version of the OHIP-G was applied [24]. This modification was performed to adapt the questionnaire to the specific objective of our study, as we wanted to evaluate the oral health-related quality of life after sinus lift procedures. Therefore, for this treatment, specific questions like “Have you had a maxillary sinusitis” were added to the questionnaire. After providing informed consent, patients completed a questionnaire, consisting of the three subcategories (1) functional limitations, (2) physical and psychological disabilities, and (3) complaints due to the surgical procedure. The implemented questions are shown in Tables 1, 2, and 3. Responses were made on an ordinal 4-point adjectival scale (0=never, 1=occasionally, 2=fairly often, and 3=very often). OHRQoL is described by summary scores of the asked items. Higher scores imply a stronger negative influence on OHRQoL; in contrast, lower scores indicate better OHRQoL. The valuation periods were divided into “pre-operative” and “recently” for subcategories (1) and (2). For subcategory (3), valuation periods were classified into “pre-operative,” “post-operative,” and “recently.”

**Table 1:** Mean value and standard deviation for the subcategory functional limitations

Item	Mean ± SD before sinus lift	Mean ± SD after sinus lift	*p* value
Have you had difficulty chewing any foods?	1.6 ± 1.2	0.4 ± 0.7	<0.001
Have you had to avoid eating some foods?	1.1 ± 1.2	0.3 ± 0.7	<0.001
Have you felt that your sense of taste was impaired?	0.4 ± 0.8	0.1 ± 0.5	<0.001
Have you had trouble pronouncing any words?	0.6 ± 0.9	0.2 ± 0.6	<0.001
Have you been unable to brush your teeth properly?	0.6 ± 0.8	0.3 ± 0.6	<0.001
Have you felt that your breath has been stale?	0.7 ± 1.0	0.4 ± 0.6	<0.001

**Table 2:** Mean value and standard deviation for the subcategory physical and psychological disabilities

Item	Mean ± SD before sinus lift	Mean ± SD after sinus lift	*p* value
Have you felt tense because of problems with your teeth, mouth or dentures?	1.8 ± 1.1	0.8 ± 1.0	<0.001
Have you felt bad because the appearance of your teeth has been affected?	1.2 ± 1.2	0.4 ± 0.7	<0.001
Have you avoided eating with other people?	0.4 ± 0.8	0.1 ± 0.3	<0.001
Have you been a bit irritable with other people?	0.3 ± 0.7	0.1 ± 0.4	<0.001
Have you avoided going out?	0.3 ± 0.7	0.0 ± 0.2	<0.001
Have you had problems managing your daily routine?	0.4 ± 0.8	0.1 ± 0.4	<0.001
Have you been unable to work to your full capacity?	0.4 ± 0.8	0. 1 ± 0.4	<0.001
Have you had difficulties to relax?	0.7 ± 1.1	0.3 ± 0.6	<0.001
Have you felt that your general health has worsened?	0.6 ± 1.0	0.2 ± 0.6	<0.001

**Table 3:** Mean value and standard deviation for the subcategory complaints due to the surgical procedure

Item	Mean ± SD pre-operative	Mean ± SD post-operative	Mean ± SD in the last time
Have you felt pain in your mouth?	0.9 ± 1.1	1.2 ± 1.0	0.3 ± 0.6
Have you had difficulties with your mouth opening?	0.2 ± 0.6	0.5 ± 0.9	0.1 ± 1.0
Have you had painful gums?	0.9 ± 1.0	1.0 ± 1.1	0.5 ± 0.8
Have you had a sore or infected jaw?	0.7 ± 0.9	0.7 ± 0.9	0.3 ± 0.7
Have you had headaches?	0.5 ± 0.9	0.5 ± 0.8	0.4 ± 0.7
Have you had ostealgia?	0.3 ± 0.7	0.6 ± 1.1	0.2 ± 0.5
Have you had pain in your maxillary sinus?	0.4 ± 1.0	0.5 ± 0.8	0.1 ± 0.5
Have you had a maxillary sinusitis?	0.3 ± 0.7	0.3 ± 0.6	0.2 ± 0.5
Have you had swellings in your mouth?	0.4 ± 0.7	0.9 ± 0.9	0.2 ± 0.5
Have you had numbness in your mouth?	0.2 ± 0.5	0.5 ± 0.9	0.2 ± 0.6
Have you had poor taste in your mouth?	0.6 ± 0.8	0.5 ± 0.8	0.2 ± 0.5

### Statistics

The Kaplan–Meier survival function was applied for the description of survival rates. To examine the statistical difference between survival rates, a log-rank test was used. Implant-related data were calculated. For statistical comparison of the paired questions and the total scores, a Wilcoxon test was applied. The intention of this study was descriptive, exploratory without a primary hypothesis. Consequently, we show descriptive *p* values of tests and no adjustment to multiple testing was done. The analyses were conducted using SPSS version 20.0 (IBM, USA).

## Results

### Survival analysis

**Fig. 2:**
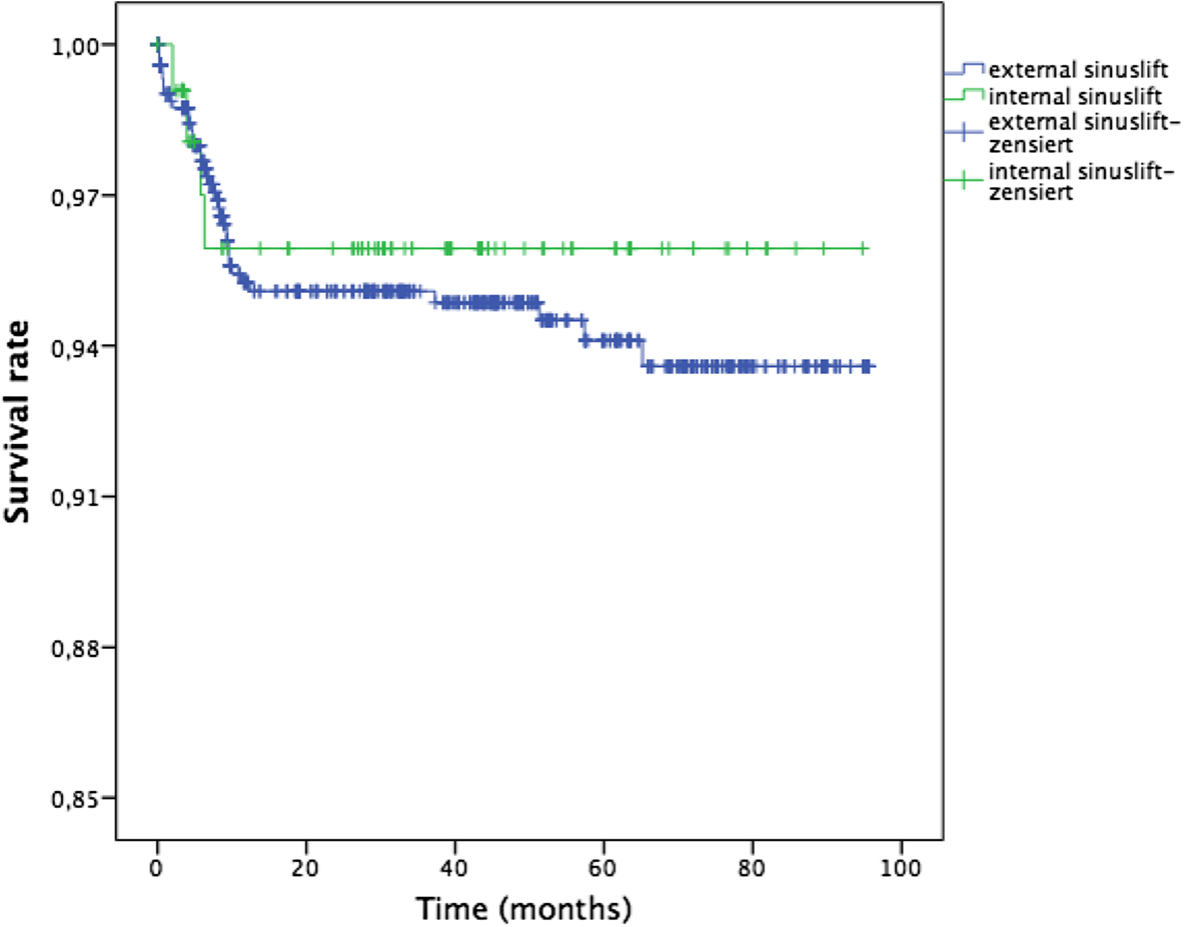
Cumulative survival rate according to Kaplan–Meier and sinus augmentation procedure

After an average time in situ of 41.2 ± 27 months (3.4 years; range 0–96 months), 40 of the 863 implants were lost. These results indicated an in situ rate of 95.4%. One-year and five-year survival rate according to Kaplan–Meier were 95.4 and 94.4%. In patients receiving an external sinus lift an in situ rate of 95.1% and in patients with an internal sinus lift an in situ rate of 96.4% after the mean follow-up of 3.4 years was achieved. These results indicated a higher survival rate for the internal sinus lift procedure, but this difference was not statistically significant (*p* = 0.614, Fig. 2). The in situ rates were 100% for implants with a length <10 mm, 95.3% for implants with a length 10–13 mm, and 93.9% for implants with a length >13 mm. These differences were not statistically significant (*p* = 0.657). Implant survival for implant diameter <3.6 mm were 100%, for implant diameter 3.6–4.5 mm 96.0%, and for implant diameter >4.5 mm 92.2%, indicating a not statistically significant difference (*p* = 0.123). For patients that were available for clinical follow-up examination, the plaque index showed that 86.6% of implants had a satisfactory degree of oral hygiene (grades 0 and 1). Concerning the gingival index, 76.4% of the implants showed a gingival index grade 0, 19.7% a gingival index grade 1, and 3.8% a gingival index grade 3. A probing depth of less than 3.5 mm at all four measured sites around each implant was determined for 82.8% of the implants. The width of keratinized mucosa was <1 mm in 38.9% of the implants, between 1 and 2 mm in 37.6% of the implants, and >2 mm in 11.4% of the implants. No keratinized mucosa was found in 12.1% of the cases.

### Pre- and post-treatment assessment of oral health-related quality of life

In 182 patients, pre- and post-treatment oral health-related quality of life after sinus augmentation procedure using a standardized questionnaire was evaluated. Subcategories for this evaluation were (1) functional limitations, (2) physical and psychological disabilities, and (3) complaints due to the surgical procedure.

Concerning functional limitations, all posed questions showed significant better values for OHRQoL after sinus augmentation procedure than before the treatment (*p* < 0.001; Table 1). The total score is calculated from the sum of the respective questions with high values indicating worse OHRQoL. The maximum total score achievable in the subcategory functional limitations was 18. Median total scores in the category functional limitations were 4.64 ± 4.3 (range 0–17) before and 1.65 ± 2.4 (0–13) after the treatment, indicating a significant difference (*n* = 169; *p* < 0.001).

In the subcategory physical and psychological disabilities, all questions had significant better values after the sinus lift (*p* < 0.001; Table 2). The total score achievable in this category was 27. Mean total scores were 5.79 ± 6.4 (range 0–27) before and 1.94 ± 3.2 (range 0–21) after the sinus augmentation procedure, indicating a significant difference (*n* = 164; *p* < 0.001).

**Fig. 3:**
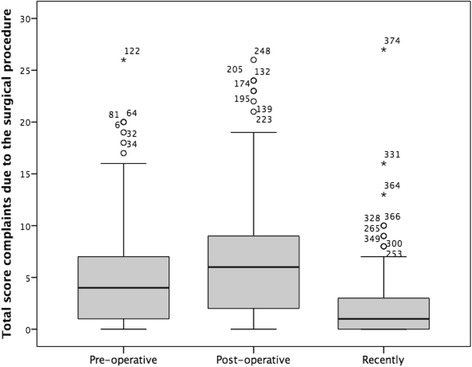
Total score for complaints due to surgical procedure pre-operative, post-operative, and recently

In the subcategory complaints due to the surgical procedure, the patients were asked to answer the items regarding the periods “pre-operative,” “post-operative,” and “recently.” Six of the 11 items (items 1, 2, 6, 8, 9, and 10) were significant worse “post-operative” compared to “pre-operative” (*n* = 126; *p* ≤ 0.03; Table 3; Fig. 3). However, comparing the periods “pre-operative” and “recently,” items 1, 3, 4, 6, 7, 8, 9, and 11 showed a significant improvement (*n* = 126; *p* ≤ 0.002). Comparison of the periods “post-operative” and “recently,” all items were significant better in the period recently (*n* = 126; *p* < 0.001). Mean total scores were 5.1 ± 5.4 (range 0–26) pre-operative, 6.9 ± 6.1 (0–31) post-operative, and 2.4 ± 3.7 (range 0–27) recently. This meant a significant difference between “pre-operative” vs. “post-operative” (*n* = 126; *p* = 0.003), “pre-operative” vs. “recently” (*n* = 126; *p* < 0.001), and “post-operative” vs. “recently” (*n* = 126; *p* < 0.001).

### Impact of implant indication on oral health-related quality of life

**Fig. 4:**
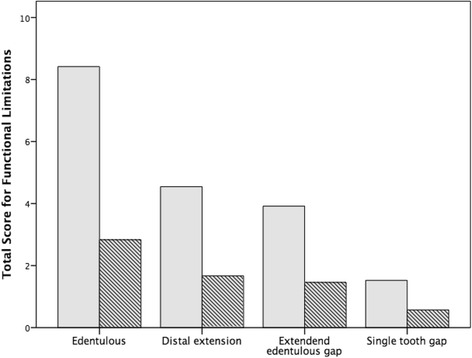
Total score for functional limitations before (*gray*) and after (*hatched*) sinus augmentation according to indications

**Fig. 5:**
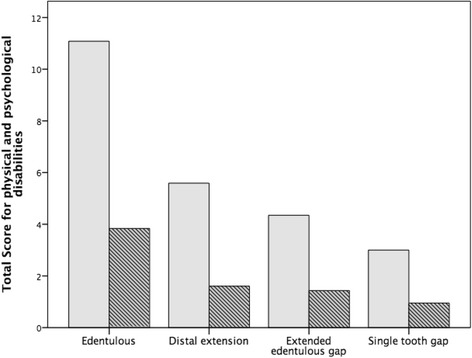
Total score for physical and psychological disabilities before (*gray*) and after (hatched) sinus augmentation according to indications

In edentulous patients, median total scores in the category functional limitations were 8.4 ± 4.1 before and 2.7 ± 2.4 after the treatment, indicating a significant improvement (*p* < 0.001; Fig. 4). In addition, patients with a distal extension situation (4.6 ± 4.0 vs. 1.7 ± 2.7; *p* < 0.001), an extended edentulous gap (3.9 ± 3.8 vs. 1.4 ± 1.9; *p* = 0.009) and a single tooth gap (1.5 ± 2.2 vs. 0.6 ± 1.3; *p* = 0.034) showed significant lower mean total scores after the rehabilitation compared to before the treatment. Concerning the category physical and psychological disabilities, mean total score of edentulous patients showed the most distinct improvement after the procedure (11.1 ± 8.2 vs. 3.7 ± 4.5; *p* < 0.001; Fig. 5). For patients with a distal extension situation (5.5 ± 5.8 vs. 1.8 ± 3.3; *p* < 0.001), with an extended edentulous gap (4.4 ± 4.9 vs. 1.4 ± 1.7; *p* = 0.007) and with a single tooth gap (3.0 ± 3.0 vs. 1.0 ± 1.2; *p* = 0.005), total score were significantly lower after the sinus lift.

## Discussion

The clinical and radiological outcomes of sinus augmentation procedures have been published in several studies [1, 3, 6, 7]. However, little data on the physical and psychological impact of this procedure on the patient is available yet. The present study evaluated pre-operative and post-treatment OHRQoL self-assessment scores of patients treated with dental implants after sinus augmentation procedures.

The one-year and five-year survival rates of the investigated implants were 95.4 and 94.4%. These results are in accordance with the recent literature. In a current meta-analysis, mean implant survival rates were 98.6 ± 2.6% for sinus augmentation procedures using bone substitute materials alone, 88.6 ± 4.1% for sinus augmentation procedures using bone substitute materials mixed with autologous bone, and 97.4 ± 2.2% for sinus augmentation procedures using autologous bone alone [1]. The mean follow-up of the investigated studies was 39.7 ± 34.6 months with a range from 4 to 170 months. Corbella et al. showed in a recent systematic review a survival rate from 95.4 to 100% after 3-year follow-up for internal sinus lift and a survival rate from 75.57 to 100% for external sinus lift [5]. Del-Fabbro et al. estimated a mean weighted cumulative implant survival at 1, 2, 3, and 5 years as 98.12, 97.40, 96.75, and 95.81% [6].

In the present study, OHRQoL after sinus augmentation was investigated using a modified version of the G-OHIP. The results showed significant better values for all three subcategories after the treatment, indicating a remarkable benefit for the patients. Concerning the influence of implant indication, edentulous patients showed the most distinct improvement after the procedure. So far, many studies have examined the quality of life in patients treated with dental implants [25–28]. However, to our best knowledge, studies investigating quality of life after sinus augmentation are very rare. Mardinger et al. examined the patient’s perception of immediate post-operative recovery after sinus-floor augmentation [29]. In this prospective study, health-related quality of life questionnaire was given to 76 patients evaluating patient perception of recovery in the four areas pain, oral function, general activity, and other symptoms. The results showed that average and maximal pain peaked on post-operative day 1 and improved on post-operative days 4 and 5. Difficulty in mouth opening was greatest on post-operative day 1 and improved on post-operative day 3. Swelling peaked on post-operative day 2 and improved on post-operative day 5. The authors concluded that an average patient undergoing sinus augmentation procedure should expect recovery within 5 days. In a prospective cohort study, Reisine et al. examined quality of life changes among post-menopausal women getting dental implants with bone augmentation procedures using OHIP-14 questionnaire [30]. The results showed that patients’ quality of life improved continuously from the pre-treatment to the 9-month assessment. Type of augmentation procedure had no significant influence on quality of life. Better et al. included 18 patients in a prospective clinical study to investigate patient’s perception of immediate post-operative recovery after sinus augmentation, using a minimally invasive implant device [31]. The minimally invasive implant device consisted of a self-tapping implant which contained an L-shaped internal channel allowing the introduction of liquids through the implant body and into the maxillary sinus. The results showed that patients’ perceptions of post-operative symptoms in the tested areas pain, oral function, general activity, and other symptoms were mostly scored “not at all” or “very little” on post-operative day 1, indicating a minimum discomfort through this procedure for the patient. In a prospective non-randomized clinical trial, changes in OHRQoL and health-related quality of life (HRQoL) after bone graft harvesting for dental implants with respect to the donor site were examined [32]. Therefore, autologous bone grafts were harvested in 23 patients either from an intra-oral or an extra-oral donor site, followed by implant placements. OHRQoL was analyzed using the OHIP-49, HRQoL was measured using the short-form 36. In the results, bone harvesting from an extra-oral donor site deteriorated HRQoL substantially more compared with intra-oral donor sites. OHRQoL impaired from baseline to first follow-up in both groups; however, changes were not statistically significant. The authors concluded that in clinical decision-making regarding donor site for bone graft harvesting, patients and clinicians should consider expected decrease in HRQoL if deciding to use extra-oral donor sites. Therefore, the authors recommended to prefer intra-oral donor sites whenever possible. In a recent study of Nickenig et al., OHIP-G 21 was evaluated in 8689 patients with various kinds of indications (free end gap, posterior single-tooth gap, anterior single-tooth gap, dental gap, and edentulous jaw) for dental implants [33]. Comparable to our results, the results showed an improved OHRQoL for all indications after prosthetic reconstruction. The modification of our OHIP score complicates the comparability of the baseline results of the mentioned study with our results. However, also patients with edentulous jaws and patients with an anterior single-tooth gap benefited most significantly from the treatment.

In order to measure OHRQoL in the present study, a specific and shortened questionnaire based on the validated and reliable OHIP score was developed to consider representative impairments of maxillary sinus augmentation like sinusitis and to relieve the clinical application. In a cross sectional study, Allen et McMillan proofed that a shortened OHIP-14 version showed a similar ability to assess OHRQoL compared to the detailed OHIP-49 version [34]. However, there has been some concern that the short-form OHIP-14 may not detect improvements following clinical intervention due to floor effects [35]. The OHIP was used as a measure because it showed high test-retest reliability and was validated in numerous cross-sectional population studies [36].

## Conclusions

Within the limitations of this study, the results demonstrated a high long-term survival for sinus augmentation procedures and significant improvement of OHRQoL after this procedure. Therefore, sinus augmentation procedures are highly valuable treatment options in implant dentistry.
